# Supplementary figures and images for: PrrA modulates Mycobacterium tuberculosis response to multiple environmental cues and is critically regulated by serine/threonine protein kinases
Source: PLoS Genet. 2022 Aug 1;18(8):e1010331. doi: 10.1371/journal.pgen.1010331 (PMC9371303; doi:10.1371/journal.pgen.1010331)

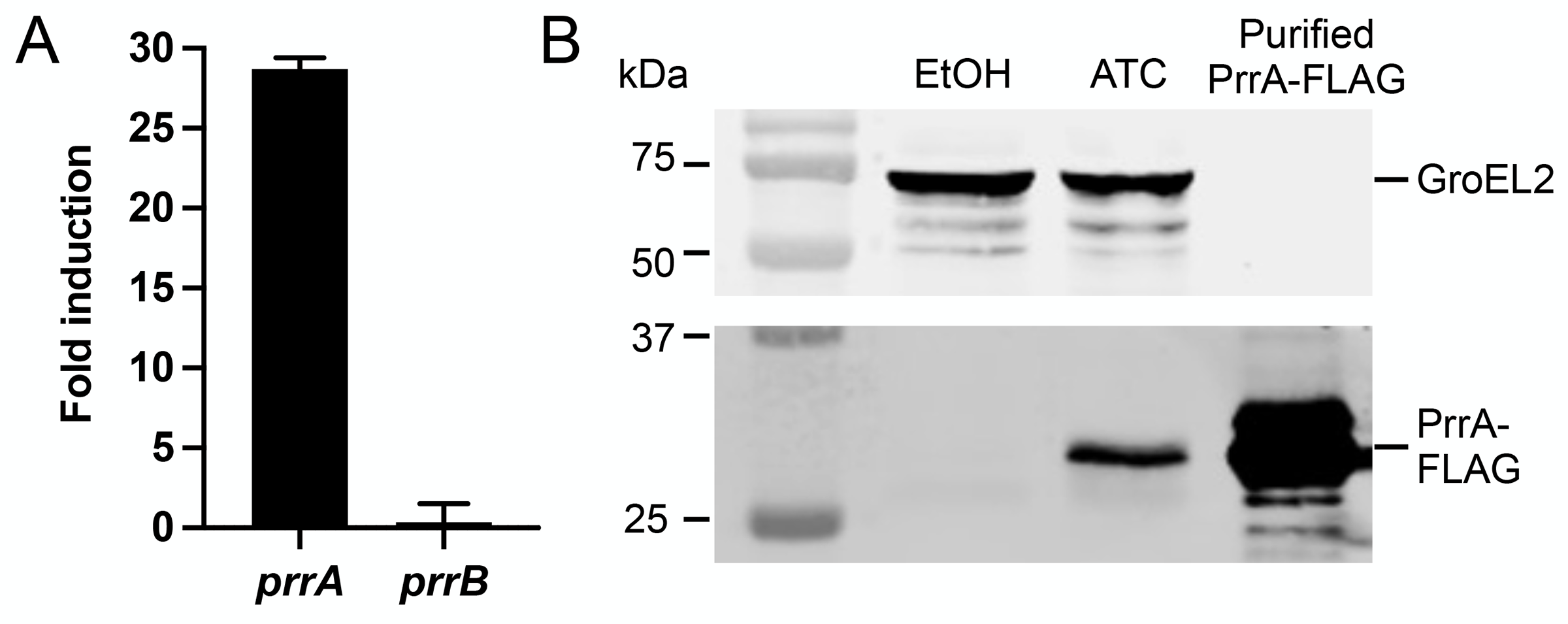

Supplement: S1 Fig — (A) qRT-PCR of Mtb(P1’::prrA-FLAG-tetON) treated with 0.1% ethanol (EtOH) or 200 ng/ml ATC for 6 hours in 7H9, pH 7.0 media. Fold change compares the ATC to the EtOH treatment. sigA was used as the control gene, and data are shown as means ± SD from 3 technical replicates. (B) Mtb(P1’::prrA-FLAG-tetON) was exposed to 0.1% EtOH or 200 ng/ml ATC for 3 days. Cultures were then normalized to the lowest OD600 and lysates analyzed by western blot. Membranes were blotted with either an anti-GroEL2 antibody as a loading control (top panel) or an anti-FLAG antibody (bottom panel). Purified recombinant PrrA-FLAG protein was used as a positive control. Blot is representative of 3 experiments. (TIF) [file pgen.1010331.s001.tif]

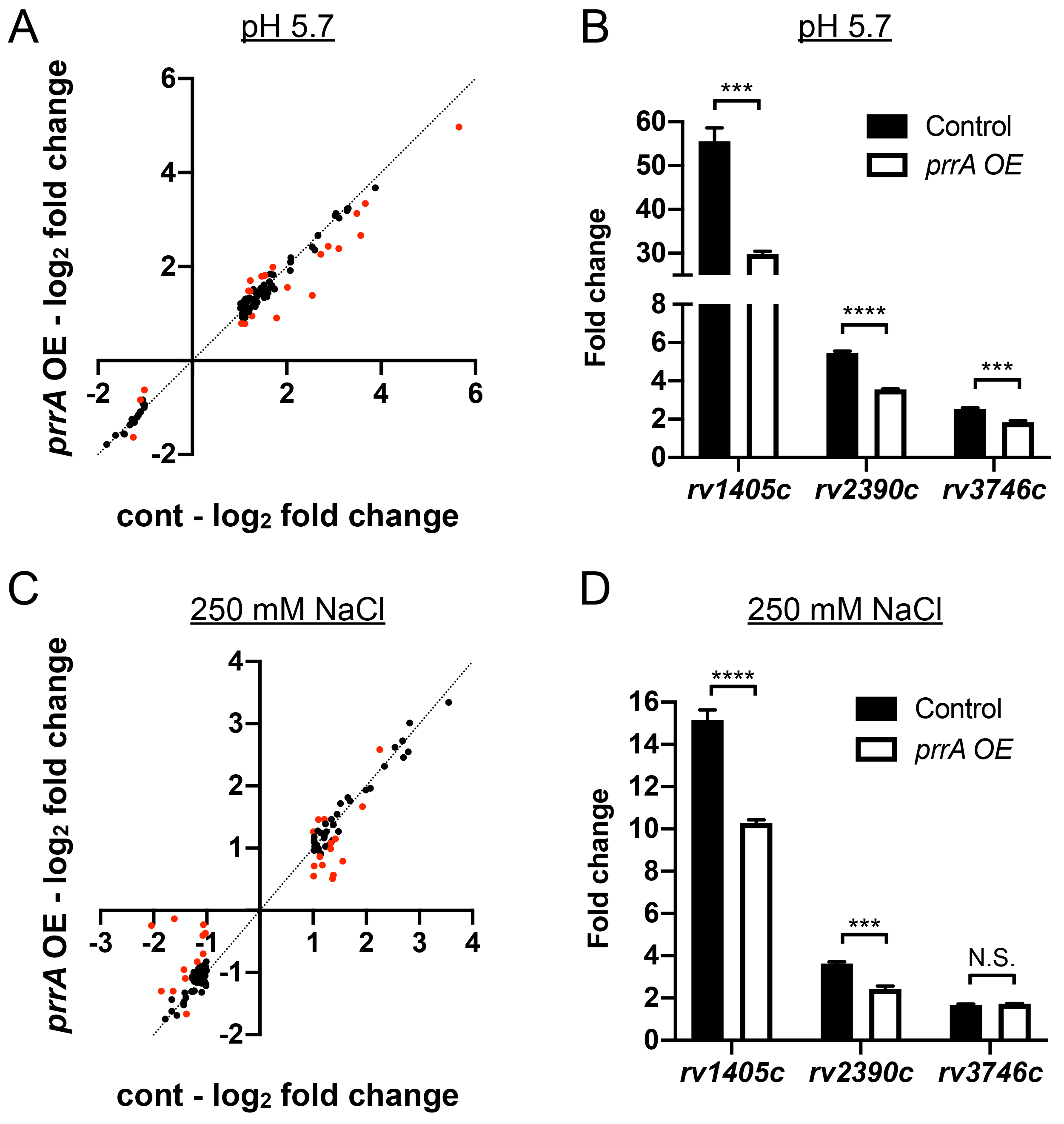

Supplement: S2 Fig — (A and B) prrA overexpression alters Mtb response to acidic pH. Mtb(P1’::prrA-FLAG-tetON, rv2390c’::luciferase) was grown in 7H9, pH 7.0 media and treated with 0.1% EtOH or 200 ng/ml ATC for 2 hours, before exposure to 7H9, pH 7.0 or 7H9, pH 5.7 for four hours, in the continued presence of EtOH or ATC as appropriate. RNA was extracted for RNA sequencing (A) or qRT-PCR (B) analysis. In (A), log2-fold change compares gene expression in the 7H9, pH 5.7 condition versus the 7H9, pH 7 control condition for each of the EtOH (“cont”) or ATC (“prrA OE”) treatment sets. Genes marked in red had a log2-fold change difference ≥0.25 between the ATC and EtOH treatment sets (p<0.05, FDR<0.01 in both sets, with log2-fold change ≥1 in the EtOH set). In (B), fold change compares gene expression in the pH 5.7 versus the control pH 7.0 condition for each of the EtOH (“control”) or ATC (“prrA OE”) treatment sets. sigA was used as the control gene, and data are shown as means ± SD from 3 technical replicates. p-values were obtained with an unpaired t-test. *** p<0.001, **** p<0.0001. (C and D) prrA overexpression alters Mtb response to high [Cl-]. Mtb(P1’::prrA-FLAG-tetON, rv2390c’::luciferase) was grown in 7H9, pH 7.0 media and treated with 0.1% EtOH or 200 ng/ml ATC for 2 hours, before exposure to 7H9, pH 7.0 ± 250 mM NaCl for four hours, in the continued presence of EtOH or ATC as appropriate. RNA was extracted for RNA sequencing (C) or qRT-PCR (D) analysis. In (C), log2-fold change compares genes expression in the 7H9, pH 7.0 + 250 mM NaCl condition versus the 7H9, pH 7.0 control condition for each EtOH (“cont”) or ATC (“prrA OE”) treatment sets. Genes are marked in red as in (A). In (D), fold change compares gene expression in the 250 mM NaCl versus the control pH 7.0 condition for each of the EtOH (“control”) or ATC (“prrA OE”) treatment sets. Data are shown as in (B). p-values were obtained with an unpaired t-test. N.S. not significant, *** p<0.001, **** p<0.0001. (TIF) [file pgen.1010331.s002.tif]

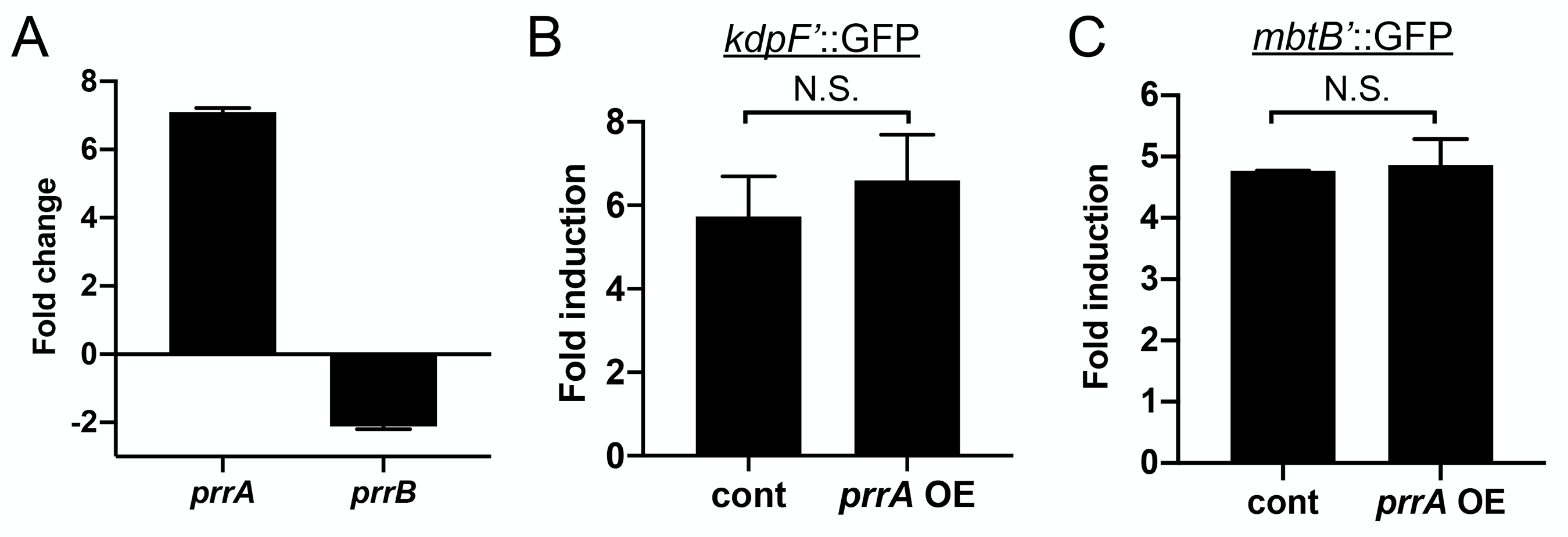

Supplement: S3 Fig — (A) prrA is inducibly overexpressed in a Mtb strain carrying a chromosomal copy of an ATC-inducible prrA-FLAG construct. qRT-PCR of Mtb(P606’::prrA-FLAG-tetON) treated with 0.1% EtOH or 200 ng/ml ATC for 6 hours in 7H9, pH 7.0 media. Fold change compares the ATC-treated condition to the EtOH control. sigA was used as the control gene, and data are shown as means ± SD from three technical replicates. (B) prrA overexpression does not affect Mtb response to low [K+]. Mtb(P606’::prrA-FLAG-tetON, kdpF’::GFP) was treated with 0.1% EtOH (“cont”) or 200 ng/ml ATC (“prrA OE”) for 24 hours before exposure to 7H9, pH 7.0 or K+-free 7H9, pH 7.0 media for 9 days. EtOH or ATC was maintained as appropriate throughout the exposure. Reporter GFP fluorescence was measured by flow cytometry, and fold induction is in comparison to the control 7H9, pH 7.0 condition. Data are shown as means ± SD from three experiments. p-value was obtained with an unpaired t-test. N.S. not significant. (C) prrA overexpression does not affect Mtb response to low iron. Mtb(P606’::prrA-FLAG-tetON, mbtB’::GFP) was treated with 0.1% EtOH (“cont”) or 200 ng/ml ATC (“prrA OE”) for 24 hours before exposure to iron-depleted media + 150 μM Fe(NO3)3 (iron-replete) or iron-depleted media + 100 μM 2,2’-dipyridyl (an iron chelator) for 9 days. EtOH or ATC was maintained as appropriate throughout the exposure. Reporter GFP fluorescence was analyzed and data presented as in (B), with fold induction in comparison to the iron-replete condition. p-value was obtained with an unpaired t-test. N.S. not significant. (TIF) [file pgen.1010331.s003.tif]

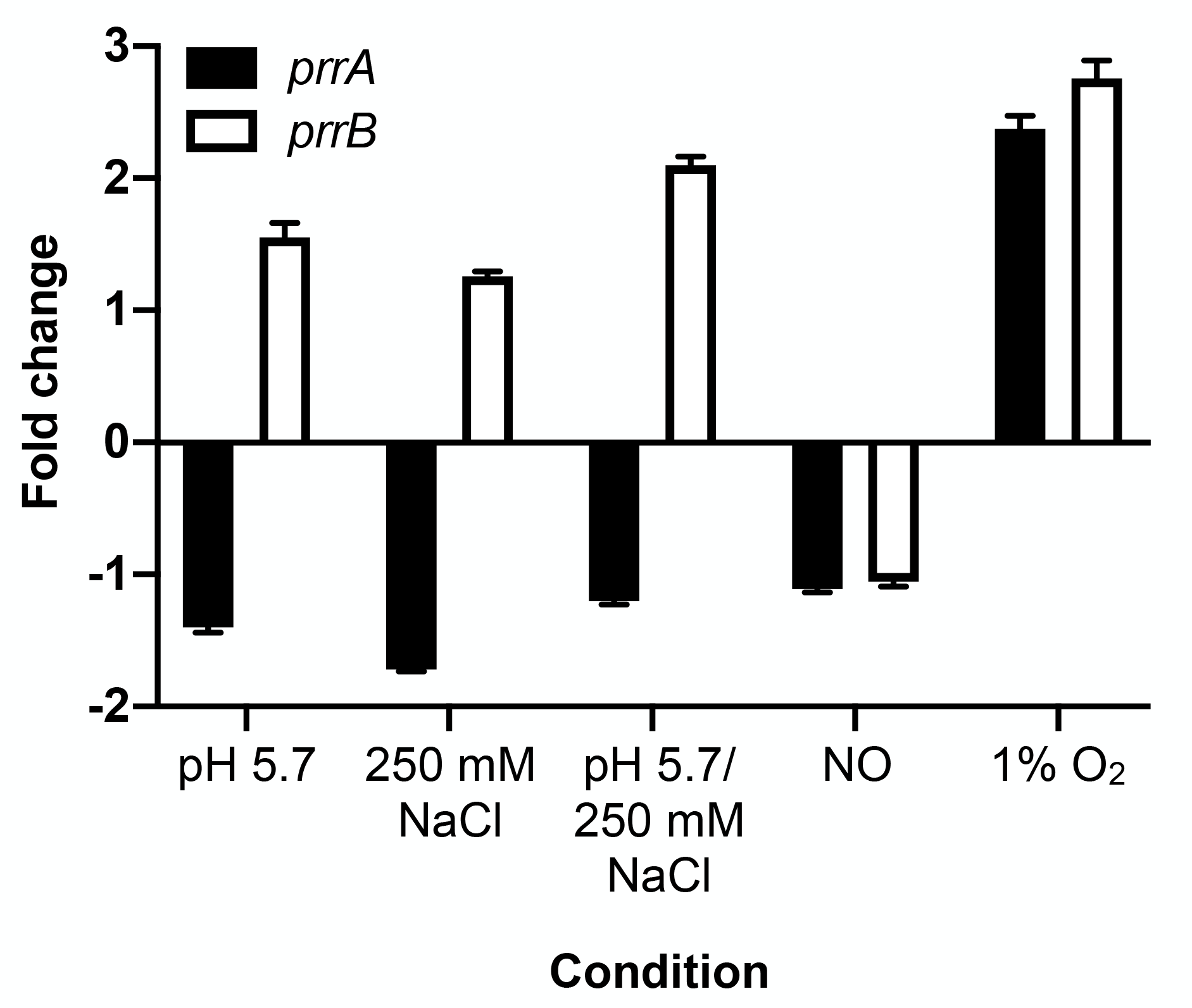

Supplement: S4 Fig — WT Mtb was exposed to each indicated environmental condition or 7H9, pH 7.0 (control) for 4 hours. qRT-PCR data is shown, with fold change comparing each environmental condition to the 7H9, pH 7.0 control condition except for the “1% oxygen” condition, where fold change is in comparison to the aerated 7H9, pH 7.0 condition at the 0 hour time point. sigA was used as the control gene, and data are shown as means ± SD from three technical replicates. (TIF) [file pgen.1010331.s004.tif]

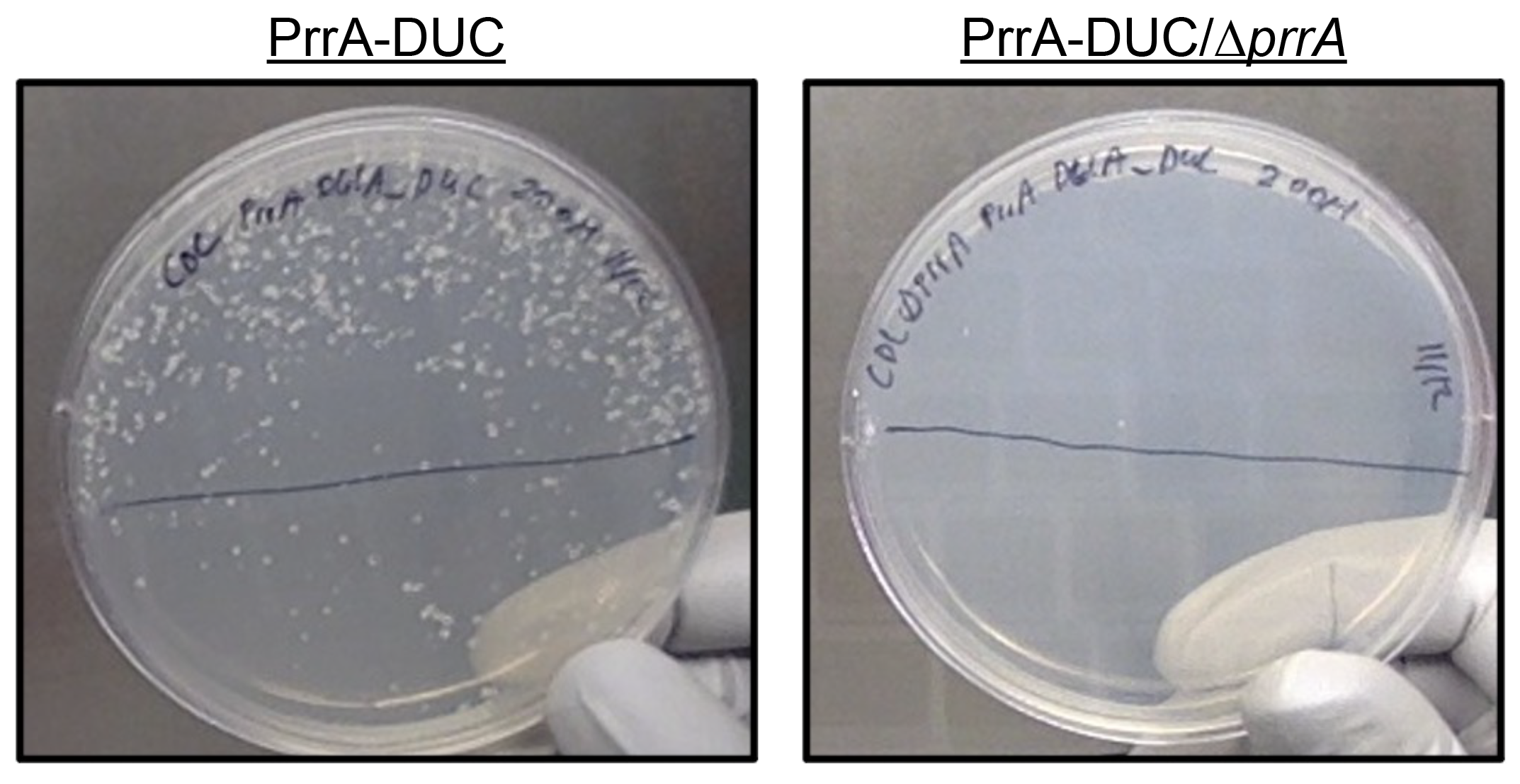

Supplement: S5 Fig — Allelic exchange of Mtb strains carrying two copies of prrA (“PrrA-DUC”) or one copy of prrA (“PrrA-DUC/ΔprrA”) was attempted, to replace the WT PrrA-DUC copy with a copy containing the PrrA-D61A variant. Only when the native copy of prrA was present were colonies obtained (left panel). (TIF) [file pgen.1010331.s005.tif]

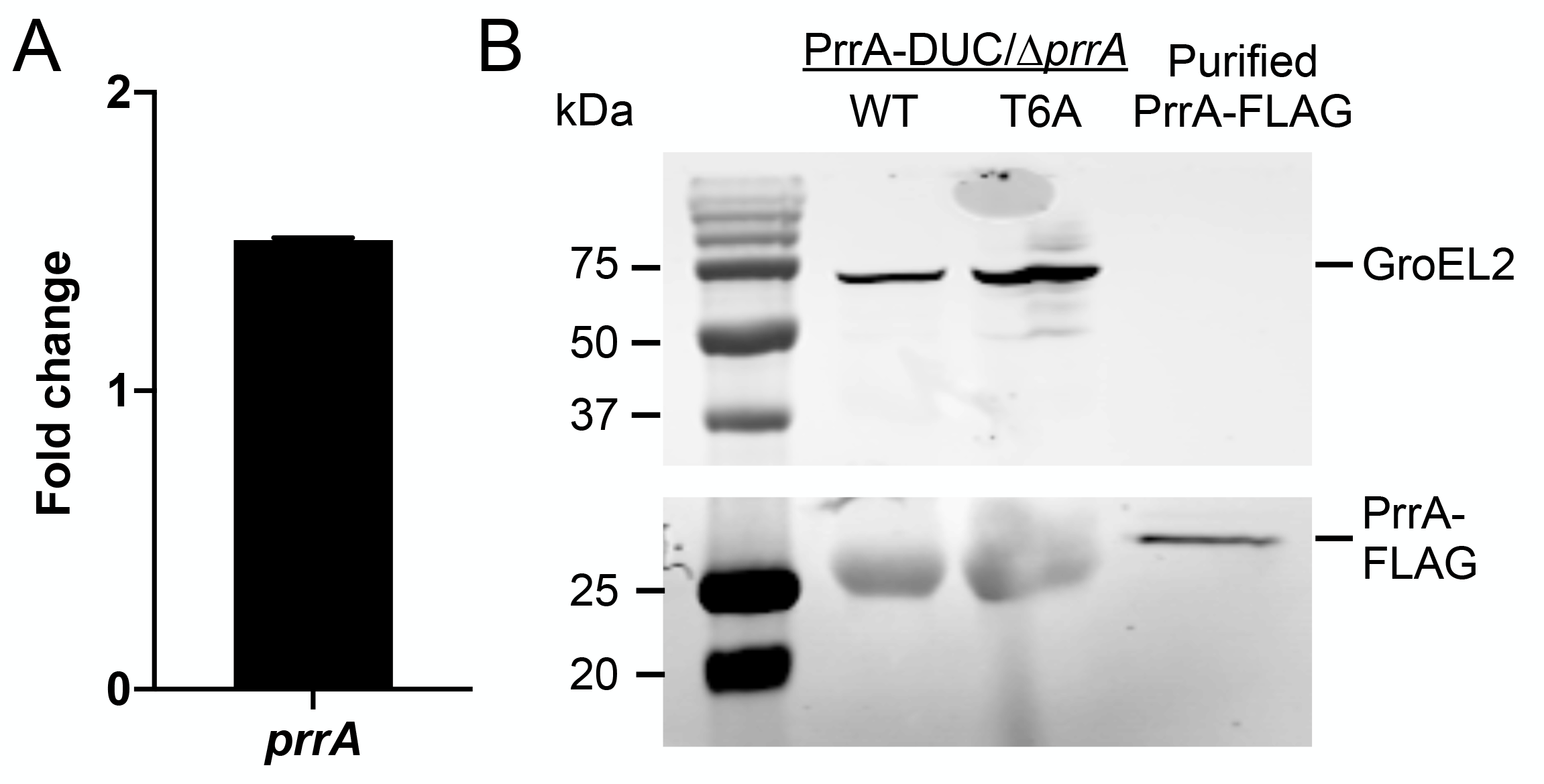

Supplement: S6 Fig — (A) shows qRT-PCR of PrrA-DUC/ΔprrA and PrrA-T6A-DUC/ΔprrA Mtb grown in 7H9, pH 7.0 media for 4 hours. Fold change compares prrA transcript levels in PrrA-T6A-DUC/ΔprrA to the PrrA-DUC/ΔprrA strain. sigA was used as the control gene, and data are shown as means ± SD from 3 technical replicates. (B) shows western blot analysis of PrrA-DUC/ΔprrA (“WT”) and PrrA-T6A-DUC/ΔprrA (“T6A”) grown in 7H9 pH, 7.0 media for 9 days, before culture was normalized to the lowest OD600 and lysates prepared. Membranes were blotted with either an anti-GroEL2 antibody as a loading control (top panel) or an anti-FLAG antibody (bottom panel). (TIF) [file pgen.1010331.s006.tif]

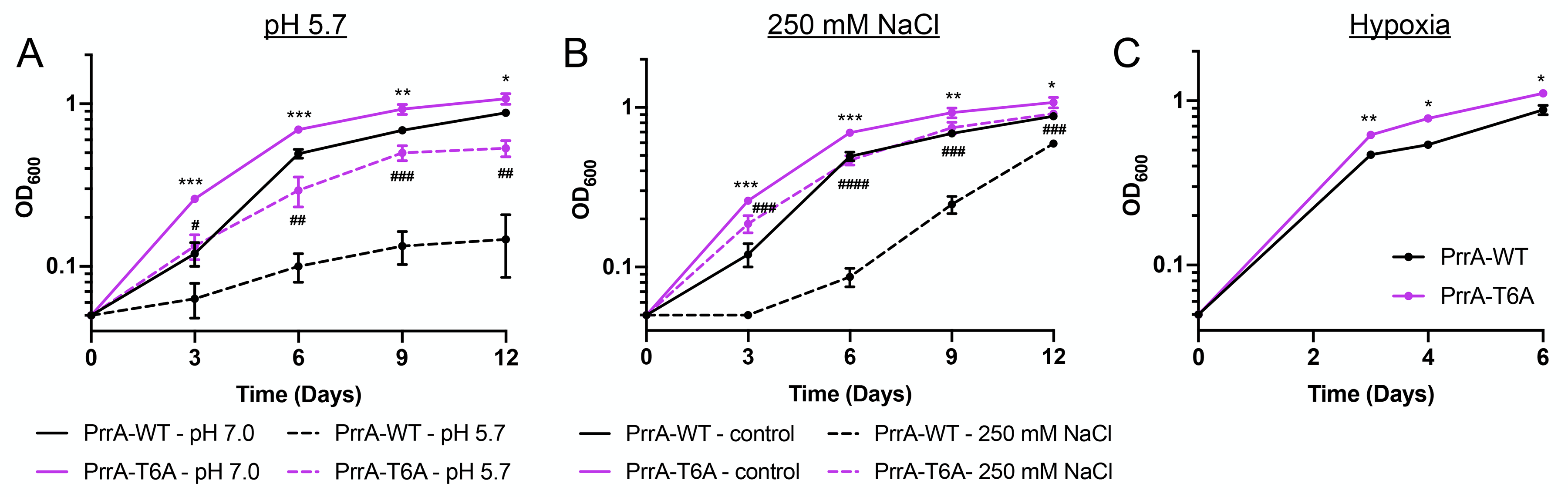

Supplement: S7 Fig — PrrA-DUC/ΔprrA (“PrrA-WT”) and PrrA-T6A-DUC/ΔprrA (“PrrA-T6A”) were grown in (A) 7H9, pH 7.0 or 7H9, pH 5.7, (B) 7H9, pH 7.0 ± 250 mM NaCl, or (C) in 1% O2 (“hypoxia”), and growth tracked over time. Data are shown as means ± SD from 3 experiments. p-values were obtained with an unpaired t-test, comparing PrrA-T6A-DUC/ΔprrA to PrrA-DUC/ΔprrA within each condition. In (A) and (B), * symbols indicate p-values for the untreated conditions, while # symbols indicate p-values for the pH 5.7 or 250 mM NaCl conditions. * p<0.05, ** p<0.01, *** p<0.001, **** p<0.0001. (TIF) [file pgen.1010331.s007.tif]

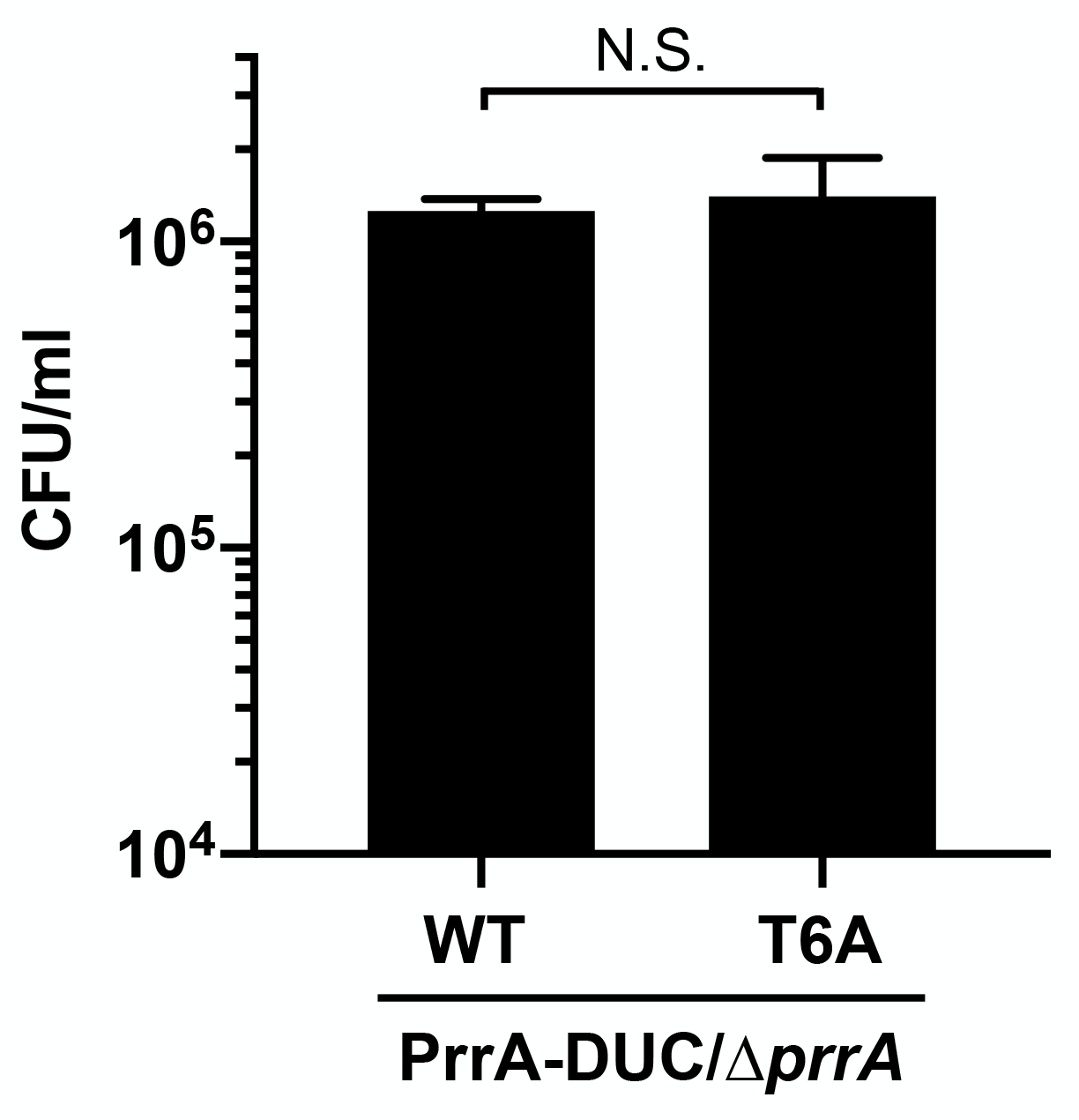

Supplement: S8 Fig — PrrA-DUC/ΔprrA ("WT") and PrrA-T6A-DUC/ΔprrA (“T6A”) Mtb input preparations for macrophage infection were plated for colony forming units (CFUs) determination. Data are shown as means ± SD from three experiments. p-value was obtained with an unpaired t-test. N.S. not significant. (TIF) [file pgen.1010331.s008.tif]
